# Artificial Intelligence and Medical Translation: An Editorial on the Ethical Considerations for Emerging Technologies in Dermatology

**DOI:** 10.7759/cureus.95350

**Published:** 2025-10-24

**Authors:** Ryan J Scheinkman, Mariana Ramirez-Posada, Sheila Sharifi, Lea Tordjman, Keyvan Nouri

**Affiliations:** 1 Dr. Phillip Frost Department of Dermatology and Cutaneous Surgery, University of Miami Miller School of Medicine, Miami, USA; 2 Department of Dermatology, Stanford University, Palo Alto, USA

**Keywords:** ai, dermatology, ethics, large language models, medical translation

## Abstract

The growing demand for medical translation services in the U.S. highlights the potential of artificial intelligence (AI), large language models (LLMs) like ChatGPT (OpenAI, San Francisco, CA), to bridge language gaps. However, their use in dermatology raises ethical concerns, including information accuracy, patient privacy, dialectical variations, legal accountability, and algorithm bias for a variety of skin colors. AI models may default to informal language, leading to misunderstandings, and their limited ability to handle less common dialects poses communication challenges. The risk of "hallucination," where incorrect information is generated, and inadequate data oversight further complicate their use. In dermatology, precise translation is crucial due to the field's visual nature and the sensitivity of cosmetic concerns. Linguistic diversity can lead to misinterpretations, affecting patient care. Dermatologists must consider these ethical implications to ensure AI tools address the nuances of dermatological terminology and regional language differences, ultimately improving patient outcomes.

## Editorial

Estimates indicate that over 25 million Americans have limited language proficiency, which has augmented the need for medical translation services [1]. Despite this increasing demand, the availability of translators, particularly for less commonly spoken languages, is restricted, and costs are high [2]. To bridge this gap, artificial intelligence (AI) may be implemented as a potential solution for translating patient instructions and serving in place of human translators during patient encounters. AI large language models (LLMs), such as ChatGPT (OpenAI, San Francisco, CA), leverage extensive online datasets to provide real-time medical translation. However, this introduces several ethical concerns, including information accuracy, patient privacy, dialectical variations, legal implications, and potential bias. In this editorial, we aim to highlight ethical considerations regarding AI translation for dermatological applications, directed at clinicians and researchers who may employ these tools.

Language variation is an important consideration, particularly regarding regional dialects and degrees of formality. In medical settings, formal language is necessary to maintain professionalism. AI models trained on large online datasets might default to less formal language, leading to potential misunderstandings and perceived unprofessionalism [2]. For example, when translating English text into Spanish, an inadequately formal translation might be interpreted as too casual, using vernacular phrases instead of technical terminology [3]. This could be deemed disrespectful to the patient and may lead to insensitivities, particularly in the setting of serious medical conditions. Additionally, less common dialects are likely underrepresented in AI training datasets, limiting AI’s ability to accommodate dialectical variations for patients speaking these dialects. Other translation services, such as Google Translate (Google, Inc., Mountain View, CA), which is a Neural Machine Translation (NMT) that evolved with AI as LLM models became available, likely face similar concerns as more recent LLMs.

A phenomenon known as “hallucination” in LLMs can result in these systems generating incorrect information [4]. Those hallucinations are categorized into two types, the intrinsic and extrinsic hallucinations, where the generated output is either unfaithful to the provided source or cannot be corroborated with the source, respectively. Hallucinations are either nonsensical or unfaithful to the provided source content [4]. This poses a considerable risk in medical translation applications, where providing patients with false information or healthcare providers with an inaccurate understanding of patient histories can have critical consequences [2]. Patient privacy concerns are also paramount, as many LLMs lack business associate agreements (BAAs) necessary for proper oversight of data usage and storage [5].

AI translation in dermatology has unique implications, particularly because of the inherent visual nature of the field, which requires precise interpretation of images and descriptions for accurate diagnosis. When AI systems translate dermatologic information, there is the risk that crucial visual contexts are overlooked, potentially leading to errors in diagnosis or treatment. Additionally, dermatology often involves cosmetic concerns, a highly sensitive topic for patients. The use of AI translation in these encounters could inadvertently violate patient privacy, as personal details might be shared or saved in AI tools. This underscores the critical concern of Health Insurance Portability and Accountability Act of 1996 (HIPAA) Privacy Rule violations in all AI-related medical applications. The HIPAA Privacy Rule establishes national standards to safeguard protected health information (PHI) by defining specific limits and conditions for its use and disclosure. It governs how healthcare providers, insurers, and related entities can use and disclose PHI while ensuring patients’ rights to access and control their own health data. The rule balances the need for information sharing in healthcare delivery with strong safeguards for patient privacy. The FDA provides guidance for AI/machine learning (ML)-enabled medical devices, emphasizing lifecycle oversight, transparency, bias mitigation, and the use of predetermined change control plans (PCCPs) so algorithm updates can occur without full re-submission when within bounds. However, HIPAA still firmly applies, meaning that AI tools used by covered entities or business associates must only access or disclose PHI for permitted purposes (treatment, payment, operations), apply the “minimum necessary” standard, and implement safeguards against unauthorized use.

Linguistic diversity presents a significant challenge for AI translation systems in medical contexts. In Spanish-speaking countries, for instance, dermatological terminology can vary widely. Words like "mancha" (spot) and "grano" (pimple) may hold different connotations or be understood differently depending on the region or country of origin, for which human translators could potentially distinguish compared to AI [2]. Similar challenges were observed in Hindi translations of radiology reports generated by ChatGPT, which were found to be unsuitable for effective patient communication due to contextual nuances [6]. Misinterpretations could lead to incorrect treatments or delayed care for communities that may not be well-represented.

These challenges extend beyond errors in translation, as AI could potentially affect the therapeutic relationship and quality of care for vulnerable populations. For patients, especially those already vulnerable due to language barriers, mistranslations could lead to confusion and erode trust in healthcare providers. Dermatologists must be mindful of the ethical implications of using AI for translation, considering risks such as mistranslation, dialectical variation, privacy issues, and misinformation, as well as legal accountability caused by misdiagnosis and wrong treatment. It is crucial to ensure that AI translation tools are equipped to address the specific nuances of dermatological terminology and regional language differences to mitigate these risks and improve patient outcomes. The performance of future LLM versions will indicate whether existing limitations are being addressed. Otherwise, targeted efforts will be required to enhance training datasets and methodologies to improve translation accuracy in capturing dermatological terminology and regional linguistic nuances. 
